# (−)-Homosalinosporamide A and Its Mode of Proteasome Inhibition: An X-ray Crystallographic Study

**DOI:** 10.3390/md16070240

**Published:** 2018-07-19

**Authors:** Michael Groll, Henry Nguyen, Sreekumar Vellalath, Daniel Romo

**Affiliations:** 1 Center for Integrated Protein Science Munich at the Department Chemie, Technische Universität München, Lichtenbergstrasse 4, 85747 Garching, Germany; 2 Department of Chemistry, Texas A&M University, College Station, TX 77843, USA; henry.nguyen@bhge.com; 3 Department of Chemistry & Biochemistry, Baylor University, Waco, TX 76798, USA; Sreekumar_Vellalath@baylor.edu

**Keywords:** beta-lactone, anticancer natural products, yeast 20S proteasome, aldol-lactonization

## Abstract

Upon acylation of the proteasome by the β-lactone inhibitor salinosporamide A (SalA), tetrahydrofuran formation occurs by intramolecular alkylation of the incipient alkoxide onto the choroethyl sidechain and irreversibly blocks the active site. Our previously described synthetic approach to SalA, utilizing a bioinspired, late-stage, aldol-β-lactonization strategy to construct the bicyclic β-lactone core, enabled synthesis of (–)-homosalinosporamide A (homoSalA). This homolog was targeted to determine whether an intramolecular tetrahydropyran is formed in a similar manner to SalA. Herein, we report the X-ray structure of the yeast 20S proteasome:homoSalA-complex which reveals that tetrahydropyran ring formation does not occur despite comparable potency at the chymotrypsin-like active site in a luminogenic enzyme assay. Thus, the natural product derivative homoSalA blocks the proteasome by a covalent reversible mode of action, opening the door for further fine-tuning of proteasome inhibition.

## 1. Introduction

**Figure 1:**
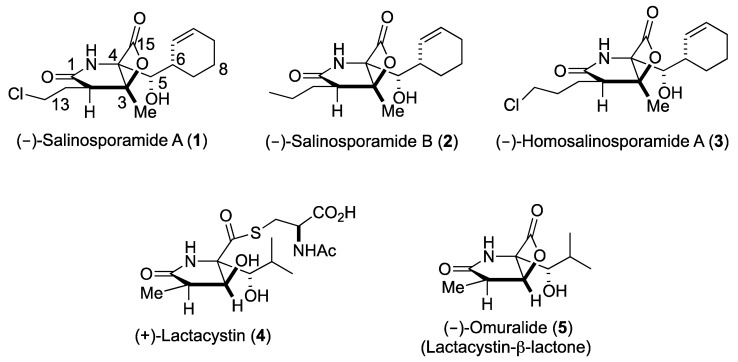
Structures of bicyclic-β-lactone proteasome inhibitors **1**–**3**, **5**, and of the precursor to **5**, (+)-lactacystin (**4**).

Upon the FDA approval of the boronic acid containing inhibitors Velcade^®^ (2003) and Ninlaro^®^ (2015), proteasome inhibitors are of enormous public interest due to their potential as anticancer therapeutics [1,2,3]. The borate pharmacophore, however, is extremely toxic and contributes to a number of severe side effects due to substantial off-target activities [4]. Hence, the number of studies on selectively and specifically inhibiting the 20S proteasome core particle (CP) has grown exponentially over the past decade and confirmed that some pathogens modulate this fascinating multi-catalytic machinery by small molecules, which have originated during evolution [5]. Thus, besides peptidyl inhibitors [6], a distinct class of proteasome ligands has emerged: small molecule β-lactone-γ-lactam natural products and their derivatives. These minimalist inhibitors have incorporated only the most essential structural features required for potent and selective CP blockage, thereby equipping their producing organisms with competitive advantages, while opening up mechanistic insights and therapeutic opportunities to humankind. The bicyclo [3.2.0] β-lactone clasto-lactacystin, also known as omuralide (**5**) and derived from the prodrug lactacystin (**4**) [7], was the first natural proteasome inhibitor that was discovered (Figure 1). While the β-lactone moiety is a moderately reactive head group, omuralide (**5**) selectively blocks the chymotrypsin-like activity of the proteasome in the nanomolar range (IC_50_ = 49 nM) [8]. X-ray analysis of the yeast (y)CP:**5** complex provided an explanation for this high degree of selectivity and potency compared to other inhibitors [9,10]. Several years later, Fenical and coworkers reported the structurally related secondary metabolites salinosporamide (Sal) A and B (**1**, **2**) from the marine actinomycete *Salinispora tropica* that block the proteasome in a similar fashion (Scheme I) [11]. Notably, SalA turned out to be a potent nanomolar inhibitor of the human proteasome (IC_50_ = 1.3 nM) and was analyzed in Phase I human clinical studies for its activity against bortezomib-resistant multiple myeloma [12]. Despite the potentially labile β-lactone, SalA also revealed promising results in mouse models towards several cancer types when administered intravenously [13]. Thus, the FDA recently approved orphan drug designation to **1** for malignant glioma, under the trade name Marizomib. In addition to superior bioactivity of **1** over its congener omuralide (35 times more potent), its unique structure, comprising five contiguous stereogenic centers, encouraged several total synthesis efforts [14], structure activity relationship studies, [15,16,17,18,19,20,21,22] biosynthetic engineering, [23,24,25,26,27] and crystallographic studies with the yeast 20S proteasome [28]. 

The enantioselective, nine-step bioinspired chemical synthesis developed by our group towards SalA utilizes a one-step route to the bicyclic fused β-lactone nucleus [29]. The key reaction involves sequential formation of the C-C and C-O bonds from a keto acid precursor via an intramolecular aldol-β-lactonization process [30,31,32]. Jacobsen had previously demonstrated the stability of a related spiro-β-lactone to various reactions [33,34], and our synthetic sequence demonstrated the feasibility of performing several reactions, including side-chain elaboration in the presence of the β-lactone ring. The β-lactone scaffold is present in several natural products as a unique proteasome inhibitory motif and we set out to prepare designed derivatives based on our synthesis of SalA [29]. Eventually, homosalinosporamide A (homoSalA, **3**) was the most compelling candidate (Figure 1) as it would allow us to assess the possibility of tetrahydropyran formation in analogy to tetrahydrofuran formation found for SalA (Scheme I). Herein, we describe an X-ray crystallographic study of the yeast 20S proteasome in complex with this ligand at 2.9 Å resolution (R_free_ = 23.3%, PDB ID 6GOP).

## 2. Results and Discussion

HomoSalA **3** was synthesized using the previously published synthetic sequence [29]. The acylation of serine derivative **6** with ketene dimer **7** furnished allyl ester **8**. Pd(0)-mediated deprotection of the allyl ester was followed by the key aldol-β-lactonization yielding the bicyclic-β-lactone (–)-**9**. Subsequently, four additional steps provided homoSalA in high optical purity (Scheme II).

The proteasome inhibitory activity of the unnatural analog, homoSalA, was measured in a luminogenic enzyme assay in comparison to SalA enabling assessment of all three active sites of yCP. Interestingly, similar to SalA, the homoSalA derivative inhibited the chymotrypsin-like activity of the proteasome in the sub-nanomolar range, while it was two orders of magnitude less potent towards the caspase- and trypsin-like activities (Table 1) [35].

**Figure 2:**
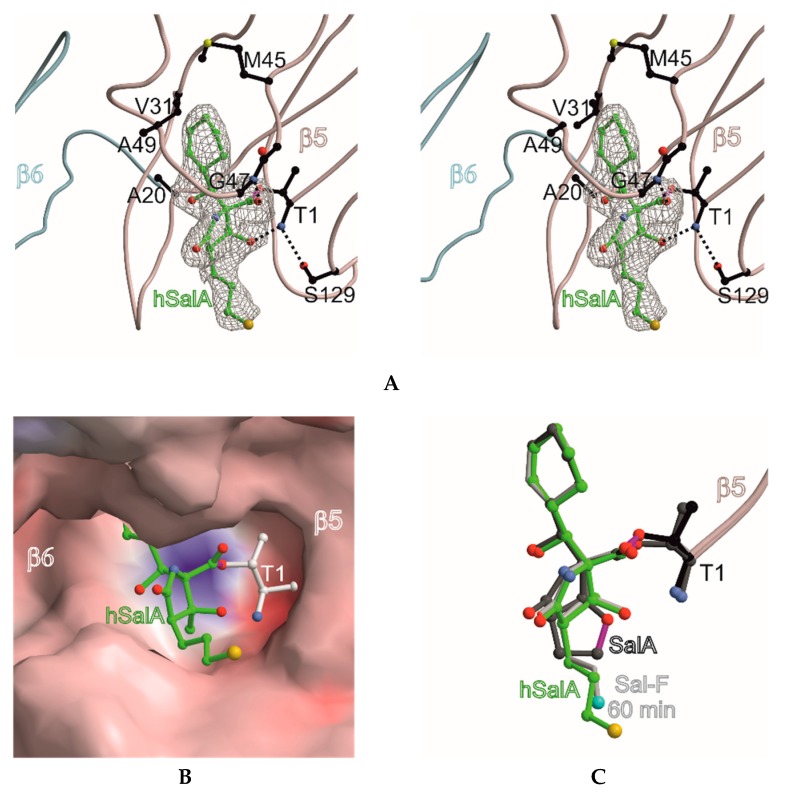
Homosalinosporamide A in complex with the yeast 20S proteasome. (**A**) Stereo view of homoSalA bound to the chymotrypsin-like active site. The backbone of subunit β5 is shown in grey and the ligand is represented in green. The 2F_O_-F_C_ electron density map for the ligand is contoured to 1σ (gray mesh). Protein residues forming the S1 specificity pocket are highlighted in black. Various sets of hydrogen bonds between homoSalA, β5 main-chain atoms and Gly47N, Thr1N, as well as Ser129O^γ^ are depicted as dashed lines; (**B**) the surface model of homoSalA bound to the active site of subunit β5; (**C**) the structural overlay of SalA (showing the tetrahydrofuran ring formed during active site inhibition), fluorosalinosporamide (Sal-F), and homoSalA bound to the active site of subunit β5.

The similar proteasome inhibition results suggest a uniform binding profile for homoSalA (**3**) and SalA (**1**). This result is interesting since it opens the possibility that the chloro-propyl moiety in homoSalA forms a tetrahydropyran ring at the proteasomal active site in analogy to the tetrahydrofuran ring observed in the yCP:SalA crystal structure (Scheme I) [10]. Therefore, we elucidated the yCP:homoSalA complex at molecular resolution to investigate its mode of action. Active yCP crystals were soaked with final compound concentrations of 5 mM for 24 h. Subsequently, diffraction data were recorded to 2.9 Å resolution (Table 2) and data processing, as well as structural refinement, were performed according to previous protocols [36,37]. The crystallographic data were refined to a final R_free_ value of 23.3% with root mean square deviation bond and angle values less than 0.007 Å and 1.1°, respectively (PDB ID 6GOP, Table 2). Inspection of the experimental 2F_O_-F_C_ electron density map displayed the natural product derivative covalently bound to the N-terminal threonine (Thr1) of all six catalytic subunits owing to the high concentrations used for crystal soaking (Figure 2). While the active site nucleophile Thr1O^γ^ opens the β-lactone ring, the positively charged Thr1NH_3_^+^-terminus acts as a Brønsted acid generating a C4-hydroxyl group from the hydrolyzed β-lactone [38]. The acyl-oxygen occupies the oxyanion hole formed by Gly47NH, even though the sp^2^-hybridized ester does not mimic a tetrahedral intermediate as observed with peptide aldehydes [39]. 

The X-ray analysis indicates that the inhibition profile of the natural product derivative is caused by the coordinative displacement of the nucleophilic water molecule from the Bürgi-Dunitz trajectory according to previous observations [28]. In particular, the ester bond joining Thr1O^γ^ to the more potent β-lactone-γ-lactam inhibitor is stabilized by C4-OH within hydrogen bonding distance to Thr1NH_2_. Moreover, the proximity of these two functional groups is maintained by the presence of the γ-lactam ring, which restricts rotation about the C-3/C-4 bond and, hence, causes a steric barrier for rapid hydrolysis by the nucleophilic water molecule. Although reformation of the β-lactone ring might be considered energetically unfavorable, Thr1NH_2_ is well positioned to catalyze this reaction, which supports the mode of action of homoSalA to act as a slow reversible CP inhibitor, similar to what is observed for SalB [10]. In contrast, SalA irreversibly blocks the CP because its chloro-ethyl moiety forms a tetrahydrofuran ring, thus, preventing deacylation of the enzyme-ligand complex [40]. Mechanistic support for successive intramolecular ring closure of SalA was outlined by crystal structures of fluorosalinosporamide (Sal-F) in complex with the yCP obtained after short and long incubation times of the ligand with yCP crystals due to the poor leaving group ability of the halogen [41]. The snapshot in subunit β5 obtained after one hour incubation revealed the inhibitor prearranged for fluoride elimination, with a well-defined side chain conformation and the CH_2_F group within van der Waals distance of the C4-O nucleophile that will introduce its displacement (Figure 2C). Thus, the position of Thr1NH_2_ within hydrogen bonding distance of C4-OH fully supports its role as a catalyst by acting, in this case, as a Brønsted base to orchestrate the reaction [38]. 

While the electron density map offered no evidence for fluoride elimination, the complex structure determined after 24 h crystal soaking time depicts complete displacement of the leaving group. These findings support a two-step reaction of Sal-F with initial, rapid formation of the acyl-enzyme intermediate, followed by the slow release of fluoride and the concomitant formation of the stable cyclic ether. Intriguingly, the X-ray structure of the yCP:homoSalA complex presented here perfectly overlays with the substituted fluorine derivative prior to cleavage. Nevertheless, the conformation of the chloro-propyl moiety is distinct from that observed for the yCP:SalB complex. In contrast, the side chain of homoSalA is stabilized by hydrophobic van der Waals interactions with the aromatic side chain of Tyr168 in subunit β5. Moreover, the crystal structure of the ligand complex reveals rotation of the chloro-*n*-propyl group from extended to gauche conformation, which is required for possible cyclization. However, while the well-defined fluoroethyl side chain requires only a modest change in the dihedral angle to achieve the final conformation of the five-membered ring, the C4-O→C-Cl distance in homoSalA is approximately 4.6 Å on average and, thus distant from the required geometry and position to form a tetrahydropyran ring system via the preferred S_N_2 trajectory (i.e., a chair-like transition state; Figure 2C). Though the CH_2_Cl-group is flexible (as indicated by high temperature factors) and free rotation is possible, the presumed short half-time of the constrained conformation likely prevents cyclization to a tetrahydropyran.

## 3. Conclusions

The chemical structures of SalA and homoSalA marginally differ by a single methylene group and their IC_50_ values are in good agreement. Yet, each compound displays a unique mode of action: whereas SalA functions as an irreversible inhibitor, homoSalA is a reversible binder. These results on the yCP:homoSalA complex structure are unexpected and demonstrate that the energetics of the secondary cyclization reaction only play a minor role in the affinity constants of SalA and homoSalA. We, therefore, conclude that the non-covalent interactions of the inhibitory P-sites with the distinct and characteristic specificity pockets is the key event in enzyme-substrate recognition. Notably, ligand binding and substrate cleavage in the proteasome strongly depend on the P1 amino acid. The chymotrypsin-like activity of the CP harbors a spacious S1-site that is particularly shaped by the hydrophobic amino acid Met45 of subunit β5. In case of homoSalA extensive hydrophobic interactions with the cyclohex-2-enylcarbinol moiety stabilize the ligand in its bound state. In contrast, only few interactions between the inhibitor’s P1-site and the S1 specificity pockets of subunits β1 and β2 are observed, resulting in significantly weaker inhibition of the caspase- and trypsin-like activities. Taken together, these detailed insights reveal that an irreversible mode of action is not necessarily required for high affinities and that reversible binding can be compensated by favorable non-covalent protein-ligand interactions, which may be exploited in future studies to expand the utility of proteasome inhibitors.

## 4. Materials and Methods

Crystals of the 20S proteasome from *S. cerevisiae* were grown in hanging drops at 20 °C as previously described [36,37] and incubated for 48 h with homoSalA at 5 mM. The protein concentration used for crystallization was 45 mg/mL in Tris(hydroxymethyl)aminomethan-HCl (10 mM, pH 7.5) and ethylenediaminetetraacetic acid (1 mM). The drops contained 1 μL of protein and 1 μL of the reservoir solution in the presence of 20 mM of magnesium acetate, 100 mM of morpholino-ethane-sulphonic acid (MES, pH 6.9), and 10% of 2-methyl-2,4-pentanediol (MPD). The space group belongs to P2_1_ with cell dimensions of a = 135.0 Å, b = 299.8 Å, c = 144.4 Å, and β = 112.6° (Table 2). Crystallographic data to a resolution of 2.9 Å for the proteasome:inhibitor complex were collected using synchrotron radiation with λ = 1.0 Å at the X06SA-beamline in SLS/Villingen/Switzerland. Crystals were soaked in a cryoprotecting buffer (30% MPD, 20 mM of magnesium acetate, 100 mM MES (pH 6.9)) and frozen in a stream of liquid nitrogen gas at 100 K. X-ray intensities were evaluated using the XDS program package [42]. The anisotropy of diffraction was corrected by an overall anisotropic temperature factor by comparing observed and calculated structural amplitudes using the program CNS [43,44]. Electron density was improved by averaging and back transforming the reflections 10 times over the two-fold non-crystallographic symmetry axis using the program package MAIN [45]. Conventional crystallographic rigid body, positional, and temperature factor refinements were carried out with CNS using the yeast 20S proteasome structure as a starting model [38]. Modelling experiments were performed with the program MAIN with current crystallographic values of R_cryst_ = 0.197, R_free_ = 0.233 [46] (Table 2).

## Figures and Tables

**Scheme I:**
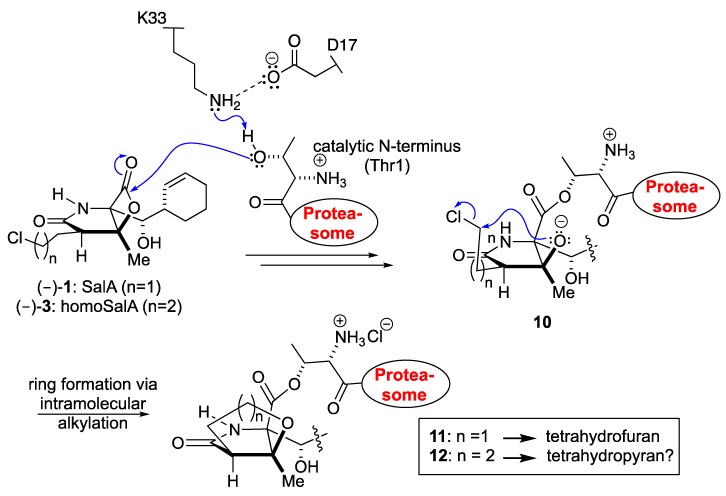
Proposed mechanism of 20S proteasome inhibition with the natural product SalA and its derivative homoSalA.

**Scheme II:**
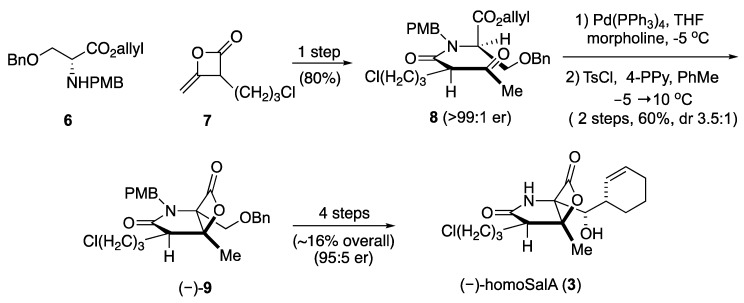
Total synthesis of homoSalA [29].

**Table 1:** Inhibition of the yeast 20S proteasome by SalA and homoSalA in a luminogenic enzyme assay.

Compound	ChT-L Activity 20S Proteasome (IC_50_, nM)	C-L Activity 20S Proteasome (IC_50_, nM)	T-L Activity 20S Proteasome (IC_50_, nM)
(–)-SalA (**1**)	0.8 ± 0.08	111 ± 22	39 ± 7
(–)-homoSalA (**3**)	0.7 ± 0.04	144 ± 12	118 ± 28

ChT-L: chymotrypsin-like activity; C-L: caspase-like activity; T-L: trypsin-like activity.

**Table 2:** Data collection and refinement statistics.

	yCP:HomoSalA
**Data Collection**	
Beamline	X06SA, SLS
Wavelength (Å)	1.0
Space group	P2_1_
Cell dimensions	
*a*, *b*, *c* (Å)	135.0, 299.8, 144.4
α, β, γ (°)	90.0, 112.6, 90.0
Resolution (Å) ^a^	50–2.9 (30.0–2.9)
No. observations	682,573
No. unique reflections ^b^	208,316
*R*_merge_ (%) ^c^	8.5 (42.4)
*I*/σ (I)	13.1 (2.8)
Completeness (%)	92.1 (91.8)
Redundancy	3.2 (3.0)
**Refinement**	
Resolution (Å)	15–2.9
No. reflections working set	196,716
No. reflections test set	10,353
*R*_work_/*R*_free_ (%) ^d^	19.7/23.3
No. atoms	
Protein	50,209
Ligand	132
Water/ions/solvents	701
R.m.s. deviations ^e^	
Bond lengths (Å)	0.007
Bond angles (°)	1.1
Average B-factor (Å^2^)	46.6
Ramachandran Plot (%) ^f^	97.8/2.0/0.2
PDB accession code	6GOP

^a^ Values in parentheses of resolution range, completeness, R_merge_, and I/σ (I) correspond to high resolution shell. ^b^ Friedel pairs were treated as identical reflections. ^c^ R_merge_(I) = Σ_hkl_Σ_j_|I(hkl)_j_ − I(hkl)]|/[Σ_hkl_ I(hkl), where I(hkl)_j_ is the *j*th measurement of the intensity of the reflection hkl and <I(hkl)> is the average intensity. ^d^ R = Σ_hkl_||F_obs_| − |F_calc_||/Σ_hkl_ |F_obs_|, where R_free_ is calculated without a sigma cut-off for a randomly-chosen 5% of reflections, which were not used for structure refinement. ^e^ Deviations from ideal bond lengths/angles. ^f^ Percentage of residues in favored region/allowed region/outlier region.
